# Supplementary material for: Meta-Analysis and Gene Set Enrichment Relative to ER Status Reveal Elevated Activity of MYC and E2F in the “Basal” Breast Cancer Subgroup
Source: PLoS One. 2009 Mar 9;4(3):e4710. doi: 10.1371/journal.pone.0004710 (PMC2650420; doi:10.1371/journal.pone.0004710)
Supplement: Dataset S1 — GSEA results from first screen. The file contains a zipped archive of the HTML results of the second GSEA screen. Reports for enriched or depleted datasets may be accessed from the “index.html” link within the my_analysis.GseaPreranked.1219975950625.rpt.zip ZIP archive. Enrichment plots are available for each of the 50 most enriched and depleted gene sets. (13.32 MB ZIP) [file pone.0004710.s001.zip › my_analysis.GseaPreranked.1219975950625.rpt.zip!//CHR5Q12.html]

Details for gene set CHR5Q12[GSEA]

|  || Dataset | zSco.ERneg.to.ERpos\_collapsed |
| Phenotype | NoPhenotypeAvailable |
| Upregulated in class | na\_neg |
| GeneSet | CHR5Q12 |
| Enrichment Score (ES) | -0.5070204 |
| Normalized Enrichment Score (NES) | -1.7543525 |
| Nominal p-value | 0.002688172 |
| FDR q-value | 0.04808294 |
| FWER p-Value | 0.889 |
Table: GSEA Results Summary

  

Fig 1: Enrichment plot: CHR5Q12      
 Profile of the Running ES Score & Positions of GeneSet Members on the Rank Ordered List

  

| PROBE | GENE SYMBOL | GENE\_TITLE | RANK IN GENE LIST | RANK METRIC SCORE | RUNNING ES | CORE ENRICHMENT || 1 | FOXD1 |  |  | 1088 | 3.373 | -0.0265 | No |
| 2 | CCNB1 |  |  | 1626 | 2.823 | -0.0205 | No |
| 3 | PART1 |  |  | 1665 | 2.789 | 0.0224 | No |
| 4 | DIMT1L |  |  | 2427 | 2.237 | 0.0019 | No |
| 5 | PDE4D |  |  | 2813 | 2.033 | 0.0063 | No |
| 6 | CD180 |  |  | 3469 | 1.723 | -0.0147 | No |
| 7 | KIF2A |  |  | 4858 | 1.203 | -0.0994 | No |
| 8 | SLC30A5 |  |  | 5721 | 0.905 | -0.1494 | No |
| 9 | GCNT4 |  |  | 6927 | 0.510 | -0.2316 | No |
| 10 | LOC57399 |  |  | 9011 | -0.315 | -0.3831 | No |
| 11 | LOC653198 |  |  | 9549 | -0.591 | -0.4138 | No |
| 12 | FLJ12595 |  |  | 9562 | -0.596 | -0.4049 | No |
| 13 | PPWD1 |  |  | 9970 | -0.825 | -0.4220 | No |
| 14 | ERCC8 |  |  | 10822 | -1.407 | -0.4629 | No |
| 15 | CSPG2 |  |  | 11207 | -1.748 | -0.4631 | Yes |
| 16 | PLK2 |  |  | 11632 | -2.151 | -0.4597 | Yes |
| 17 | PDE8B |  |  | 12262 | -2.924 | -0.4590 | Yes |
| 18 | TRIM23 |  |  | 12357 | -3.034 | -0.4163 | Yes |
| 19 | MAST4 |  |  | 12400 | -3.100 | -0.3686 | Yes |
| 20 | P18SRP |  |  | 12602 | -3.442 | -0.3272 | Yes |
| 21 | CDK7 |  |  | 12756 | -3.758 | -0.2771 | Yes |
| 22 | ANKRA2 |  |  | 12870 | -4.068 | -0.2188 | Yes |
| 23 | MCCC2 |  |  | 13143 | -5.005 | -0.1572 | Yes |
| 24 | FLJ13611 |  |  | 13170 | -5.183 | -0.0741 | Yes |
| 25 | THBS4 |  |  | 13174 | -5.197 | 0.0110 | Yes |
Table: GSEA details [plain text format]

  

Fig 2: CHR5Q12: Random ES distribution      
 Gene set null distribution of ES for **CHR5Q12**
